# Cerium oxide-based nanozyme suppresses kidney calcium oxalate crystal depositions via reversing hyperoxaluria-induced oxidative stress damage

**DOI:** 10.1186/s12951-022-01726-w

**Published:** 2022-12-08

**Authors:** Jiwang Deng, Bangxian Yu, Zhenglin Chang, Sicheng Wu, Guanlin Li, Wenzhe Chen, Shujue Li, Xiaolu Duan, Wenqi Wu, Xinyuan Sun, Guohua Zeng, Hongxing Liu

**Affiliations:** 1 Department of Urology, Guangdong Key Laboratory of Urology, Guangzhou Institute of Urology, The First Affiliated Hospital of Guangzhou Medical University, Guangzhou Medical University, Guangzhou, China; 2 Department of Urology, The Second Affiliated Hospital of Guangzhou Medical University, Guangzhou Medical University, Guangzhou, China

**Keywords:** Cerium oxide nanoparticles, Oxidative stress damage, Oxalate, Reactive oxygen species

## Abstract

Oxidative stress damage to renal epithelial cells is the main pathological factor of calcium oxalate calculi formation. The development of medicine that could alleviate oxidative damage has become the key to the prevention and treatment of urolithiasis. Herein, porous nanorods CeO_2_ nanoparticles (CNPs) were selected from CeO_2_ with different morphologies as an antioxidant reagent to suppress kidney calcium oxalate crystal depositions with excellent oxidation resistance due to its larger specific surface area. The reversible transformation from Ce^3+^ to Ce^4+^ could catalyze the decomposition of excess free radicals and act as a biological antioxidant enzyme basing on its strong ability to scavenge free radicals. The protection capability of CNP_S_ against oxalate-induced damage and the effect of CNP_S_ on calcium oxalate crystallization were studied. CNP_S_ could effectively reduce reactive oxygen species production, restore mitochondrial membrane potential polarity, recover cell cycle progression, reduce cell death, and inhibit the formation of calcium oxalate crystals on the cell surface in vitro. The results of high-throughput sequencing of mRNA showed that CNPs could protect renal epithelial cells from oxidative stress damage caused by high oxalate by suppressing the expression gene of cell surface adhesion proteins. In addition, CNP_S_ can significantly reduce the pathological damage of renal tubules and inhibit the deposition of calcium oxalate crystals in rat kidneys while having no significant side effect on other organs and physiological indicators in vivo. Our results provide a new strategy for CNP_S_ as a potential for clinical prevention of crystalline kidney injury and crystal deposition.

**Supplementary Information:**

The online version contains supplementary material available at 10.1186/s12951-022-01726-w.

## Introduction

Urolithiasis is a widespread disease worldwide with regional variations in occurrence [1]. The incidence of kidney stones in adults in China was 6.4% [2]. The recurrence rate after surgery is about 30–50% within 5 years, and as high as 80–90% within 10 years [3]. Calcium oxalate stones are the most common stone type, accounting for about 80% of all kidney stones [4]. However, the specific etiological mechanism of calcium oxalate stone formation is still unclear, and there are still no effective preventive methods and therapeutic drugs.

High oxalate-induced oxidative stress damage in renal epithelial cells is a key factor in the formation of calcium oxalate stones [5–7]. Excessive reactive oxygen species (ROS) release is the main cause of oxidative stress damage [8–10]. Oxidative stress injury in cells will damage the cell membrane, mitochondrial dysfunction, cell cycle disorder, and even lead to cell death [11–15]. Antioxidants can antagonize the oxidative stress injury of renal cells caused by hyperoxaluria, reduce renal function damage, and inhibit the development of stones [16–19].

Although some progress has been made in the treatment of nephrolithiasis in recent years, its morbidity and recurrence rates remain high. At present, the clinical drug for urinary calculi mainly includes citrate-based drugs, allopurinol [20], and thiazide diuretic [21]. These drugs need to be taken for a long time, and the side effects are large, which leads to poor patient compliance and is difficult to achieve the ideal preventive effect [22]. Therefore, it is of great social significance and commercial value to develop a new type of calculus prevention and treatment drug with high antagonism stone efficiency, few adverse reactions, and good safety.

Nanozyme usually refers to artificial enzymes which present highly effective enzyme-like properties and could be widely used for anti-inflammatory, anti-oxidative damage and cancer treatments, etc. [23–29]. In recent years, studies have shown that cerium oxide nanoparticles (CNPs) present good antioxidant capacity and the ability to scavenge excess reactive oxygen species [30–34]. Different crystal forms, spatial structures and particle sizes of CNPs have different scavenging abilities of ROS [35]. The reversible cycle of Ce^3+^ and Ce^4+^ on the surface of CNPs plays a role similar to biological antioxidant enzymes, converting superoxide anion (•O^2−^) into H_2_O_2_, and further into water, so that CNPs have a strong ROS scavenging ability. The scavenging of excessively produced ROS is the key to its antioxidant effect [36]. Manneet al. [37] found that CNPs could scavenge ROS and attenuate the increase in inflammatory mediators and subsequent acute kidney injury caused by a variety of microorganisms. Wenget al. [38] found that CNPs could prevent chemotherapy-induced acute kidney injury without interfering with chemotherapy effects by regulating ROS-mediated upstream and downstream oxidative stress-related genes. These studies show that CNPs can exert their biological effects safely, non-toxic and efficiently, making them have great potential in the prevention and treatment of urinary calculi. The advantages of CNPs in the prevention and treatment of urinary calculi are as follows: 1) CNPs are widely used in the field of biomedicine and have good biosafety due to their highly efficient, no obvious toxic and side effects and green nanozyme; 2) The spatial structure of CNPs enables them to reversibly convert Ce^3+^ into Ce^4+^, which makes CNPs have strong free radical scavenging capacity, which is the key to their antioxidant capacity.

In this study, we screened a variety of CNPs with different physicochemical properties and selected porous nanorods CeO_2_ nanoparticles as an antioxidant reagent to further explore the protective effect of CNPs on oxidative damage of renal tubular epithelial cells and the inhibitory effect on calcium oxalate crystal deposition in rat kidneys. The in vitro results showed that CNPs could effectively reduce reactive oxygen species production, restore mitochondrial membrane potential polarity, restore cell cycle progression, reduce cell death, and inhibit the formation of calcium oxalate crystals on the cell surface in vitro which could significantly reduce the oxidative stress injury of renal tubular epithelial cells induced by high oxalate, reduce the adhesion of calcium oxalate salts to renal tubular epithelial cells, inhibit the formation of calcium oxalate crystals, and improve the cell state. In addition, CNP_S_ could significantly reduce the pathological damage of renal tubules and inhibit the diol-induced deposition of calcium oxalate crystals in rat kidneys. Therefore, these results show that CNPs have a significant role in inhibiting the adhesion and deposition of calcium oxalate crystals and thus antagonize the formation of calcium oxalate due to their strong ability to resist oxidative stress damage, and provide guidance for the screening of novel drugs for inhibiting crystalline kidney injury and crystal deposition.

## Experiments and methods

### Materials

Human proximal tubule epithelial cells (HK-2) were purchased from American Type Culture Collection (ATCC, Manassas, VA, USA); rat renal tubular epithelial cells (NRK-52E), rat renal interstitial fibroblast cells (NRK-49 F), Madin-Darby Canine Kidney cells (MDCK) were obtained from Cell Bank of the Chinese Academy of Science (Shanghai, China). Cell Counting Kit-8 (CCK-8) was purchased from Dojindo (#CK04, Dojindo Laboratories, Shanghai, China). Hematoxylin and Eosin (H&E) Staining Kit (#C0105M), reactive oxygen (ROS) assay kit (S0033M), JC-1 mitochondrial membrane potential assay kit (C2003S), Calcein/PI Cell Viability (C2015L) were purchased from Beyotime Biotechnology (Shanghai, China). Ultrasensitive™ S-P immunohistochemistry kit (#KIT-9730) was purchased from Maixin Biotechnology (Fuzhou, China). Cell Cycle Detection Kit (KGA512) was purchased from Nanjing KeyGen Biotech. Ethylene glycol (EG, #E808735), ammonium chloride (AC, #A801305), and sodium oxalate (Ox, #S818088) were purchased from Macklin Biochemical (Shanghai, China). Kim-1 primary antibody (#ab47635, Abcam), Catalase primary antibody (#ab209211, Abcam). TRIzol reagent (Invitrogen, CA).

### Synthesis and characterization of CNPs

Porous nanorods CeO_2_ nanoparticles (CNPs) were synthesized as the previously reported method [39]. Briefly, 1.736 g Ce (NO_3_)_3_·6H_2_O and 19.2 g NaOH were mixed in 70 mL water and refluxed at 100 °C for 24 h at normal pressures to obtain nonporous Ce(OH)_3_/CeO_2_ nanorods. Subsequently, the precipitates were subjected to a stainless steel autoclave at 160 °C temperatures for 12 h to eliminate Ce(OH)_3_.

Ceria nanopolyhedra, nanorods, and nanocubes were synthesized by changing the ratio of Ce(NO_3_)_3_·6H_2_O to NaOH as the previously reported method [40]. Briefly, 0.868 g of Ce(NO_3_)_3_·6H_2_O in 5 mL deionized water and a certain amount of NaOH (0.016 g for nanopolyhedra, 9.6 g for nanorods, and nanocubes) in 35 mL deionized water were mixed and stirring for 30 min. Subsequently, the mixture was subjected to hydrothermal treatment for 24 h at 100 °C for nanopolyhedra and nanorods, and 180 °C for nanocubes. Finally, the precipitates were obtained and dried at 60 °C.

The as-synthesis porous CeO_2_ nanorods were characterized by TEM, XPS, scanning electron microscopy (SEM), X-ray powder diffraction (XRD), and Mapping. The surface area of porous nanorods CeO_2_, Ceria nanopolyhedra, nanorods, and nanocubes were measured by BET.

### Biosecurity of CNPs in different types of kidney cells

HK-2, NRK-52E, NRK-49 F, MDCK cells were seeded into 96-well and incubated with different concentrations of CNPs (0.19, 0.38, 0.78, 1.56, 3.125, 6.25, 12.5, 25 and 50 µg/mL) for 24 h. 10 µL CCK-8 was added to each well and incubated for 1.5 h at 37 °C. The OD values were detected by a microplate reader at 450 nm to calculate the cell viability.

### Cell viability of HK-2 cells after oxalate damage

HK-2 cells were seeded into 96-well plates for 24 h. Then, different concentrations of oxalate (0.5, 0.75, 1.0, 1.25, 1.5, 2.0, 2.5 and 3.0 mM) were added into the 96-well plate for the incubation of another 24 h. 10 µL CCK-8 was added to each well and incubated for 1.5 h at 37 °C. The OD values were detected by a microplate reader at 450 nm to calculate the cell viability.

### Cell viability of HK-2 cells after being protected with CNPs

HK-2 cells were seeded into 96-well plates for 24 h. The cells were then divided into three groups: (A) Normal control group: only serum-free culture medium was added; (B) oxalate-damaged group: 1 mM oxalate dissolved in serum-free DMEM/F-12 medium incubated for 24 h; (C) CNPs protected group: different concentrations of CNPs (0.19, 0.38, 0.78, 1.56, 3.125, 6.25, 12.5, 25 and 50 µg /mL) mixed with 1 mM oxalate were dissolved in serum-free DMEM/F-12 medium and co-included with cells for 24 h. Then, the cell viability was calculated with the CCK-8 method.

### Cell morphology observation

HK-2 cells were seeded at a density of 1 × 10^5^ cells/mL. Three groups were stained with a Hematoxylin and Eosin Staining kit and observed under a light microscope at ×100 magnification.

### Intracellular reactive oxygen species (ROS) detection

HK-2 cells were seeded into 6-well plates at a density of 1 × 10^5^ cells/mL. After being protected by CNPs, the cells were washed and incubated with 1mL diluted DCFH-DA for half an hour at 37 °C. Then, the cells were washed, and removed excess DCFH-DA to observe the ROS distribution under a fluorescent microscope.

### Measurement of mitochondrial membrane potential (Δψm)

HK-2 cells were seeded into 6-well plates at a density of 1 × 10^5^ cells/mL. After the oxalate-damaged cells were protected by CNPs, the HK-2 cells were stained with 500 µL JC-1 for 1 h at 37 °C. Intracellular JC-1 distribution was observed under a fluorescent microscope.

### Cell viability detection by calcein-AM/PI staining

Cells were seeded into 6-well plates at a density of 1 × 10^5^ cells/mL. After the oxalate-damaged cells were protected by CNPs, the living cells and dead cells distribution was observed under a fluorescent microscope using a calcein/PI cell viability assay Kit.

### Cell cycle analysis by flow cytometry

Cells were seeded into 6-well plates at a density of 1 × 10^5^ cells/mL and synchronized for 12 h. After the oxalate-damaged cells were protected by CNPs, the cells were collected and fixed using 70% ethanol at − 20 °C overnight, then the treated cells were resuspended in 500 µL PI/RNase A and incubated in darkness for 30 min at RT. The count of PI-labeled DNA was analyzed by flow cytometry.

### Observation of organelles by transmission electron microscope (TEM)

Cells were seeded into cell dish at a density of 1 × 10^5^ cells/mL. After the oxalate-damaged cells were protected by CNPs, all cells were collected. Then electron microscope fixative solution was added to fix the cells overnight at 4 °C. The cell samples were further fixed, washed, dehydrated and cleaned as previously reported method [41]. Finally, the samples were embedded in EPON 812 overnight at room temperature. Then cut and stained with uranyl acetate/lead citrate to obtain ultrathin Sects. (60–80 nm) which could be visualized using a transmission electron microscope (Hitachi).

### Lactate dehydrogenase (LDH) release assay

HK-2 cells at a density of 1 × 10^5^ cells/mL were inoculated per well in 96-well plates and incubated for 24 h. The released LDH of different groups was determined according to the manufacturer’s protocol.

### RNA extraction and sequencing

The HK-2 cells were seeded as described previously and divided into the normal group, oxalate damage group and 12.5 µg/mL CNPs protected group. The supernatant was aspirated and washed 3 times with PBS, followed by adding 1 mL of Trizol reagent per 10^6^ cells, repeatedly blowing until no clumps of cells could be seen, transferring to 1.5 mL of RNase-free EP tubes and total RNA was extracted using TRIzol reagent (Invitrogen, CA). PCR amplification single-cell sequencing was done using Illumina HiSeqTM 2500 by Gene Denovo Biotechnology Co. (Guangzhou, China).

### Animal experiment

All experimental procedures were performed under protocols approved and were approved by the Animal Care Commission of the First Affiliated Hospital of Guangzhou Medical University. All rats were raised in the laboratory animal room of the First Affiliated Hospital of Guangzhou Medical University, and the rats were raised according to the feeding standard of SPF animals. All rats were maintained in autoclaved microisolator cages and provided with sterile drinking water and chow ad libitum. Rats were housed at room temperature and at 55% humidity, 12 h light-dark cycle during the experimental period. Each rat was allowed to adapt to the environment around the feeding room for a week before the study.

Male Sprague–Dawley rats (6–8 weeks old, 180–220 g) were purchased from Guangdong Medical Laboratory Animal Center and were randomly divided into the following 4 groups: Control group (n = 5), rats were allowed free access to food and water; CaOx crystallization model group (EG + AC, n = 5), at days 1–3, 15–17 of the experiment period, rats were given 2 mL of 1% (v/v) ammonium chloride (AC) by gavage daily, meanwhile free access to drinking water that contained 1% (v/v) EG for 3 weeks; different morphologies of CNPs treatment group (EG + AC + 0.5 CNPs, EG + AC + 1.5 CNPs group, n = 5), 2 mL of 0.5 and 1.5 mg/mL/kg wt CNPs solution was given by gavage daily, meanwhile free access to drinking water that contained 1% (v/v) EG for 3 weeks; Control + 1.5 CNPs group (n = 5), 2 mL 1.5 µg/mL/kg wt CNPs solution was given by gavage daily.

### In vivo biodistribution studies of CNPs in nude mice

Indocyanine green (ICG)-labeled CNPs was first synthesized by mixing ICG(1 mg/mL) solution with DSPE-PEG functioned different morphologies of CNPs overnight and centrifuge to obtain the precipitate. In vivo biodistribution studies of CNPs were obtained using an IVIS Lumina III animal living imager (PerkinElmer, USA). To visualize biodistribution of CNPs in mice, 200 µL 1.5 µg/mL/kg.wt ICG-labeled CNPs were oral gavage to a nude mouse with lavage needle. Then the mice were anesthetized (2% isoflurane in 2 l/min O_2_). The real-time imaging of ICG-labeled CNPs (1.5 µg/mL/kg wt in 200 µL of saline) in nude mice (6 week) was monitored at 0, 1, 2, 4, 8, 12 h.

### Histologic analysis

At the end of the experimental procedures, 24-h urine, serum, and tissue samples were collected from all rats and were euthanised finally. Then the tissues (heart, liver, spleen, lung and kidney) were fixed in 10% formalin solution, embedded with paraffin, and serially sectioned at 5-µm thickness for staining. Histopathological changes were observed by optical microscope (Olympus, Japan) and tissue scanner (PathScope, GEN DigiPath, USA). Pathological evaluation of the kidney tissue sections was performed with H&E staining. CaOx Crystal deposition was analyzed by polarized light optical microphotography (CX31-P, Olympus, Japan).

### Pizzolato staining

CaOx crystal deposition in kidney sections was examined through Pizzolato staining. Briefly, a mixture of 100 µL each of 30% H_2_O_2_ and 5% silver nitrate was poured onto each slide containing tissue sections. The slides were exposed to light from a 60-W incandescent lamp at a distance of 30 cm for 30 min. The slides were then washed thoroughly with PBS and counterstained with Nuclear Fast Red Staining Solution before being dehydrated. Finally, all slides were observed by an optical microscope.

### Immunohistochemical staining (IHC)

IHC followed a standardized process. Immunohistochemical staining for anti-KIM-1 (Abcam, 1:200) and anti-Catalase (anti-CAT, Abcam, 1:200) were performed on kidney sections using an Ultrasensitive TM S-P immunohistochemistry kit. Kidney sections were incubated overnight at 4 °C with anti- KIM-1and anti-CAT, then, they were incubated with biotinylated goat anti-rabbit or anti-mouse IgG and avidin-biotin-peroxidase complex (ABC). Finally, the sections were gradient alcohol dehydration, xylene transparent, and neutral gum sealing. A polarizing microscope (CX31 Olympus, Tokyo, Japan) and tissue scanner (PathScope,GEN DigiPath, NV, USA) were used to observe the kidney sections. The protein expression of each antibody was calculated by Image J software.

## Results and discussion

### Synthesis and characterization of CNPs

**Fig. 1:**
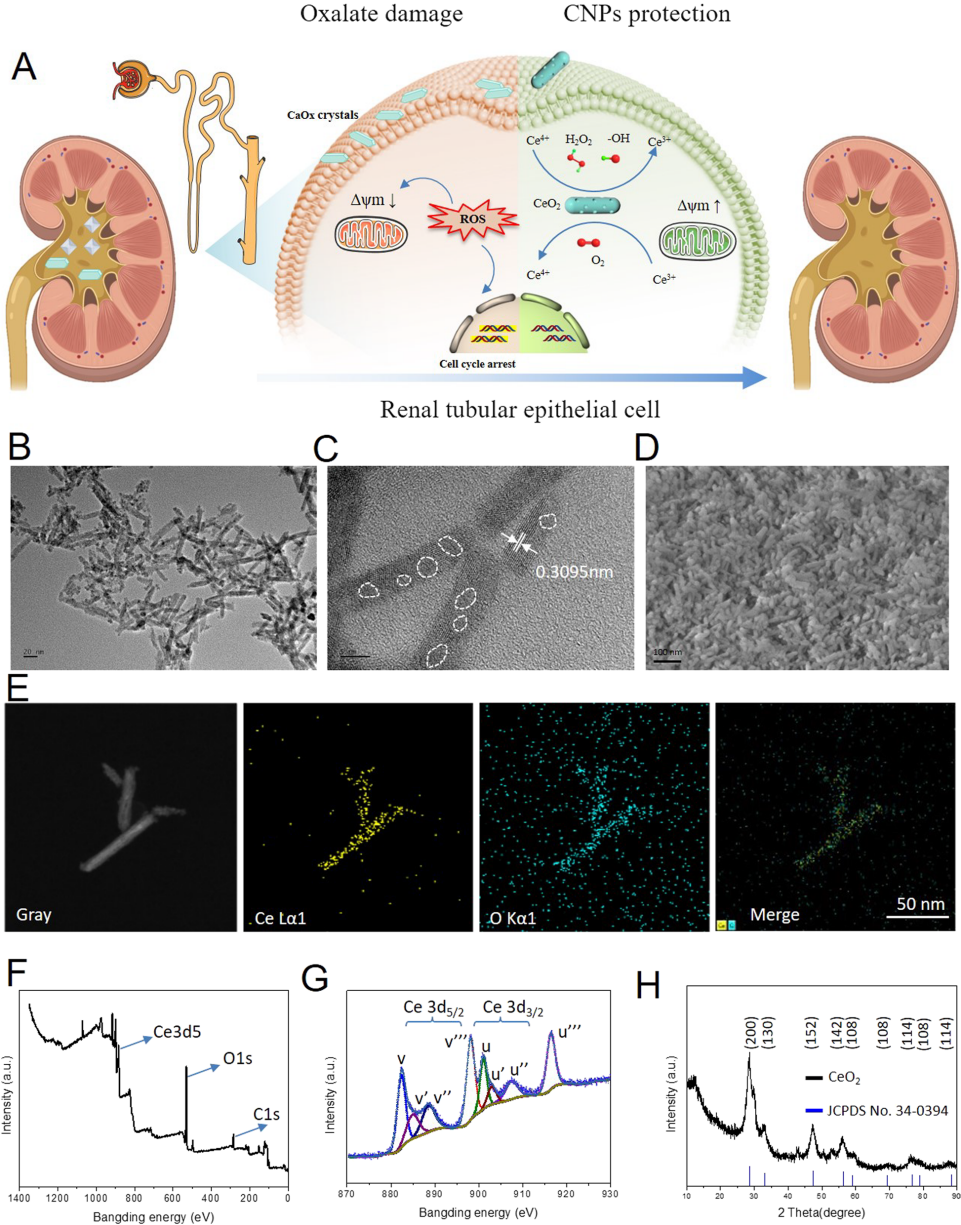
Synthesis and characterization of CNPs. **A** The illustration of the porous nanorods CNPs suppresses kidney calcium oxalate crystal depositions via reversing hyperoxaluria-induced oxidative stress damage; **B** The TEM images of CNPs; **C** High-resolution TEM images of the CNPs; **D** SEM micrographs of the CNPs; **E** STEM-EDS elemental mapping images of the CNPs; **F** XPS spectra of the CNPs; **G** XPS spectra of Ce3d_5/2_ and Ce3d_3/2_; **H** XRD analysis of the CNPs and the standard card of CeO_2_.

In this study, we hypothesize that CNPs could significantly reduce the oxidative stress injury of renal tubular epithelial cells induced by high oxalate, reduce the adhesion of calcium oxalate salts to renal tubular epithelial cells, and inhibit the formation of calcium oxalate crystals (Fig. 1A). The morphology of nano enzyme could affect the catalytic effect. For selecting the ceria nano enzyme with the best catalytic effect, four kinds of nano-cerium oxide including porous nanorods, nanorods, nanopolyhedra and nanocubes were synthesized. The XRD, XPS and FTIR of CeO_2_ with different morphologies were measured and the results in Additional file 1: Fig. S1 show that there is no significant difference between XRD, XPS and FTIR of CeO_2_ with different morphologies, which proves that CeO_2_ with different morphologies is only different in morphology and has the same composition. The surface area of the as-synthesized nano-cerium oxide was measured with BET (Additional file 1: Fig. S2A) and among which the maximum area of porous nanorods is 105.3885 m^2^/g. The ROS scavenging ability of the four kinds of nano-enzymes was also investigated and the result in Additional file 1: Fig. S2B shows that the porous nanorods nano-cerium oxide represents the best ROS scavenging effect. Therefore, porous nanorod nano-cerium oxide was selected as the best antioxidant agent in the next experiment. To verify the enzyme synthesis, we first observed its morphology using a transmission electron microscope, and the results in Fig. 1B show that the cerium oxide synthesized by the hydrothermal method is a short roller shape of a length of 50 nm; a high-resolution transmission electron microscope in Fig. 1C show that the lattice stripes are 0.3095 nm, and the surface has some pores indicating porous nanorods of the as-prepared CNPs; The scanning electron microscope results in Fig. 1D also confirmed the nanorods morphology. The results of EDS analysis in Fig. 1E show that the main elements of the synthesized rod nanomaterials are Ce and O; XPS analysis results in Fig. 1F, G show that the valence state of the synthesized nanomaterials was mainly Ce^3+^ and Ce^4+^. The results of the XRD in Fig. 1H are also consistent with the results of the standard card, and these results have shown that the mesh CeO_2_ was successfully synthesized.

### The safety and the protective effect of CNPs from oxalate on HK-2 cells

**Fig. 2:**
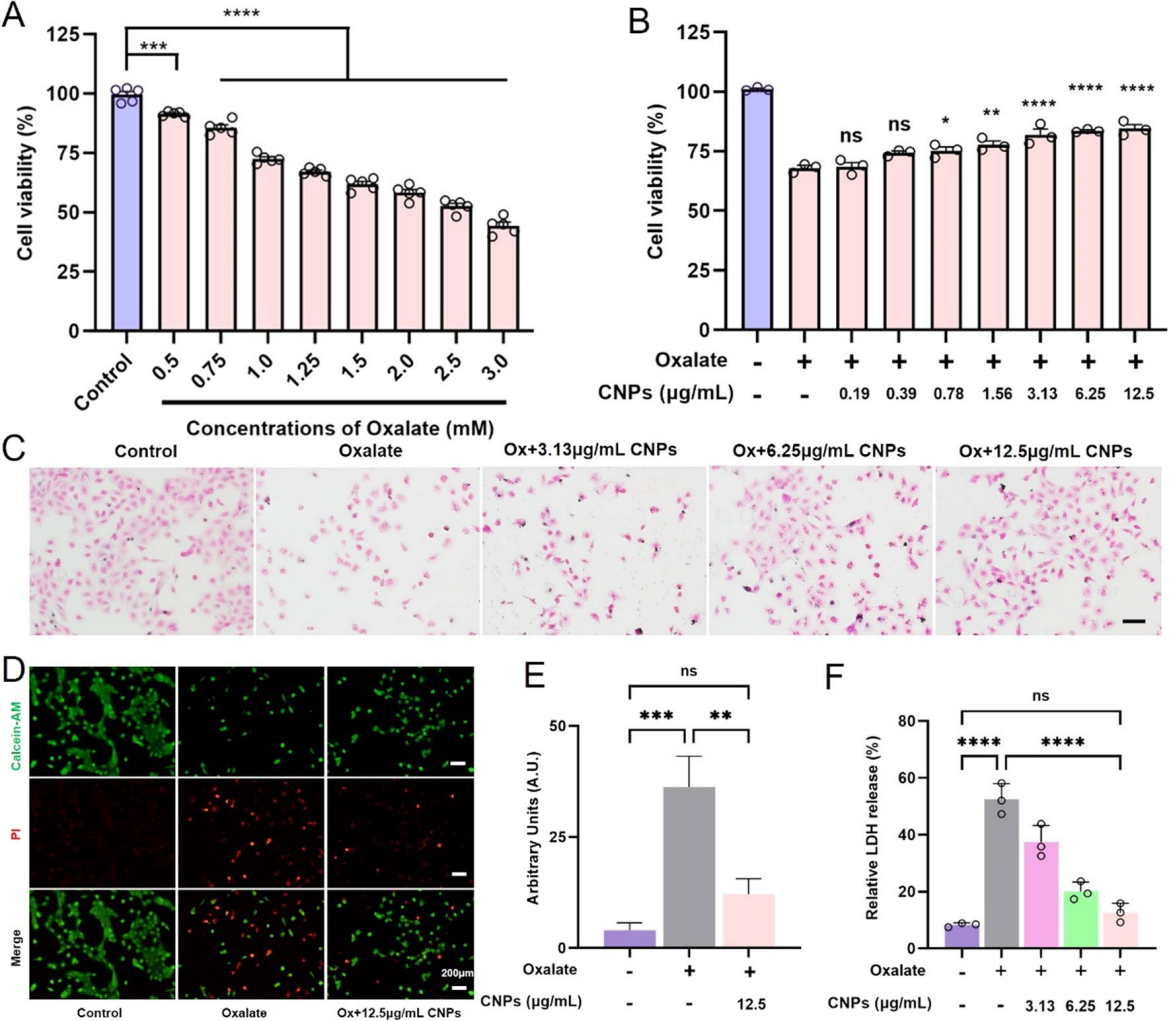
CNPs significantly inhibited HK-2 cell damage induced by oxalate and enhanced cell viability. **A** The effect of varying concentrations of oxalate solution on the viability of HK-2 cells; **B** The capacity of different concentrations of CNPs to protect injured HK-2 cells; **C** H&E stain of the HK-2 cells in different groups; **D**, **E** Calcein-AM/PI) staining and corresponding fluorescence semi-quantification **F** the released amount of LDH in different groups. CNPs protection time: 24 h; CNPs concentration: 3.125, 6.25, 12.5 µg/mL; Oxalate damage concentration: 1 mM; Oxalate damage time: 24 h; **C**, **D** magnifications were ×100, scale bar = 200 μm; * indicates P < 0.05, ** indicates P < 0.01, **** indicates P < 0.0001, ns indicates P > 0.05, no significant difference

Oxalate supersaturation is an important cause of the formation of CaOx kidney stones. HK-2 cells were exposed to different concentrations of oxalate solution, the cell viability decreased with the increase of oxalate concentration. Cell viability was negatively correlated with oxalate concentration; the higher the oxalate concentration, the lower the cell viability (Fig. 2A). 1 mM oxalate was chosen as the preferred concentration for the HK-2 cell damage model.

Then, we examined the effects of different concentrations of CNPs on the viability of the different kidney cell lines. The results showed that CNPs had no effect on the viability of four types of kidney cells at up to 50 µg/mL, indicating that CNPs has a good biosafety (Additional file 1: Fig. S3). To investigate the protective effect of CNPs on HK-2 cells, different concentrations of CNPs were used for pre-protection of HK-2 cells. When HK-2 cells were exposed to 1 mM oxalate for 24 h, the cell viability was reduced to 67.75%, whereas the cell viability was recovered by different concentrations of CNPs after 24 h protection. CNPs with a concentration of 12.5 µg/mL showed the best protection effect (Fig. 2B).

In order to observe the morphological changes of cells more visually, H&E staining was used. As shown in Fig. 2C, normal HK-2 cells were relatively homogeneous and full; while after treatment with 1 mM oxalate, the number of cells was obviously reduced, the morphology was obviously wrinkled, and a large number of tiny short rod-shaped calcium oxalate crystal particles were visible around the cells. Under the protection of CNPs, the number of cells increased obviously and the morphology was improved, and the protective effect was more obvious with the increase of CNPs concentration. In particular, the best protective effect was shown in the 12.5 µg/mL CNPs group. CNPs could reduce the damage of oxalate to HK-2 cells and inhibit the production of calcium oxalate crystals, thus improving the status of damaged cells.

The intracellular esterase activity and cell membrane integrity were detected with Calcein AM/PI. The density of HK-2 cells in the normal control group was roughly 80–90%, and the cells had good morphological outlines in large numbers and showed intense green fluorescence (Fig. 2D, E). Oxalate resulted in a significant increase in the number of red-stained cells and a significant decrease in the number of green-stained cells. After protection by different concentrations of CNPs, the red fluorescence was significantly reduced, indicating that the number of dead cells became less.

Furthermore, the released amount of lactate dehydrogenase (LDH) was also detected (Fig. 2F). After the treatment with different concentrations of CNPs, the relative released LDH was significantly reduced. 12.5 µg/mL CNPs group showed the best protection effect. CNPs could significantly inhibit cell membrane damage induced by high oxalate.

### CNPs could attenuate oxalate-induced cell oxidative injury

**Fig. 3:**
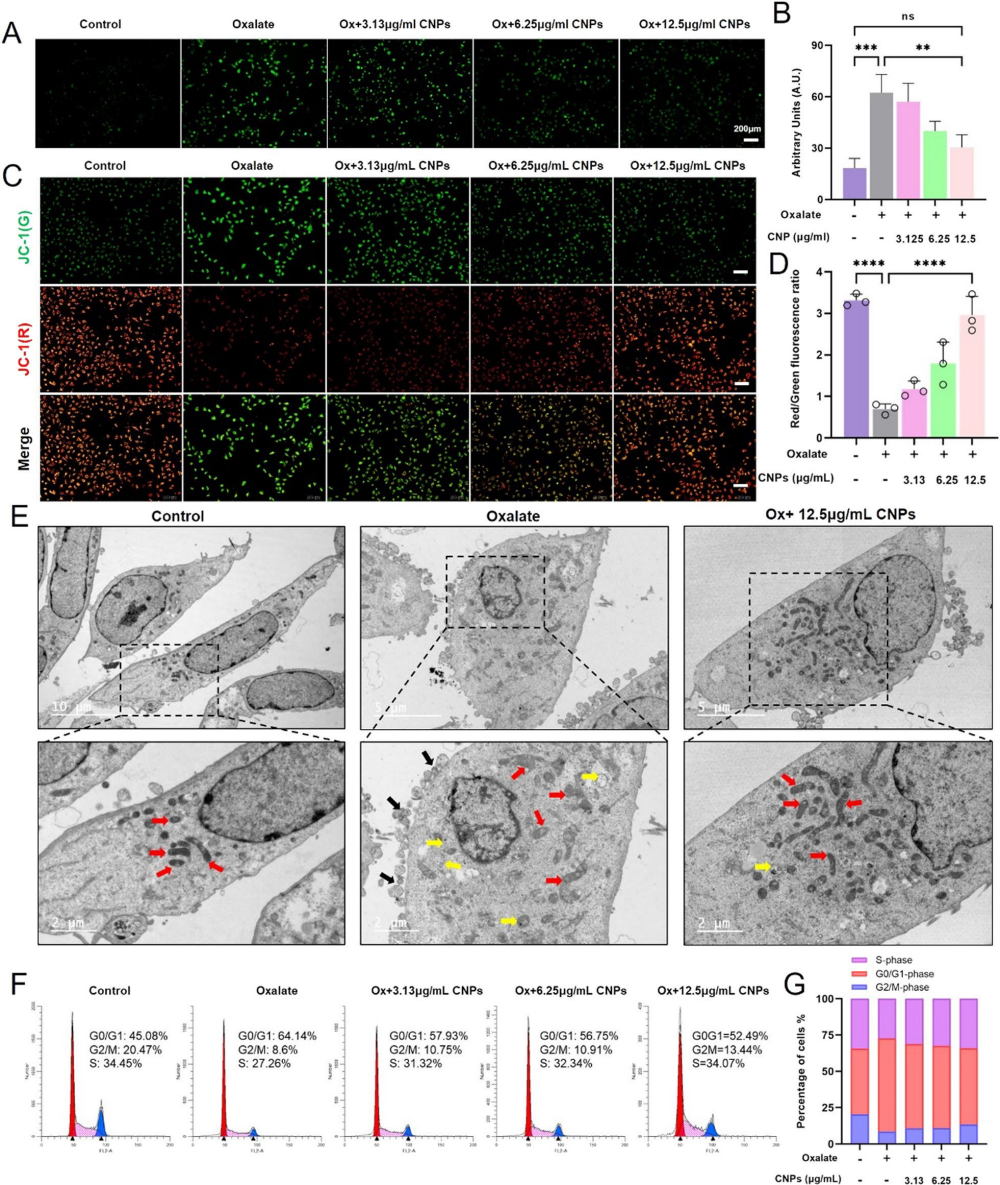
CNPs significantly inhibited HK-2 organelle damage and cell cycle arrest induced by high oxalate.** A** Detection of intracellular reactive oxygen species (ROS); **B** quantitative analysis results of the ROS in different groups. **C**, **D** Mitochondrial membrane potential in HK-2 cells and its fluorescence semi-quantification assay; **E** TEM observation of the HK-2 cells in different groups. **F**, **G** Flow cytometry detection of HK-2 cells’ cycle and quantitative percentage; Protection of HK-2 cells exposed to oxalate by 12.5 µg/mL of CNPs for 24 h, HK-2 cells were observed by TEM, the red arrows indicate mitochondria, yellow arrows indicate autophagic vacuoles, autophagosomes and autolysosomes. The black arrows indicate the distended microvilli. Oxalic acid damage concentration: 1 mM; oxalate damage time: 24 h; CNPs protection time: 24 h; CNPs concentration: 3.125, 6.25, 12.5 µg/mL. Magnification is ×100, Scale bar = 200 μm; ** indicates P < 0.01, *** indicates P < 0.001, **** indicates P < 0.0001, ns indicates P > 0.05, no significant difference

High concentrations of oxalate can induce the production of free radicals in kidney cells, thus activating mitochondria and causing stress damage. Meanwhile, excessive ROS can directly damage DNA, causing double-stranded DNA breaks, causing blockage of DNA replication, and causing cell cycle arrest, which in turn affects normal cell function and leads to the development of disease. The ROS levels were measured with a DCFH-DA probe and observed under a fluorescence microscope (Fig. 3A, B). The results showed that obvious green fluorescence was observed in the oxalate injury group, which indicated oxalate caused an obvious production of ROS. While after the protection by different concentrations of CNPs, the fluorescence intensity decreased as the concentration increased. Among them, the 12.5 µg/mL CNPs group exhibited the weakest fluorescence intensity, which showed the largest antioxidant effect. CNPs can inhibit oxidative damage in HK-2 cells to achieve cell protection.

A decrease in mitochondrial membrane potential is associated with early apoptosis. We analyzed the mitochondrial membrane depolarization of oxalate-treated and CNPs-protected HK-2 cells by staining with JC-1 fluorescent probe (Fig. 3C, D). The normal cells emitted high red fluorescence, whereas oxalate exposure caused a decrease in red fluorescence intensity, and emitted high green fluorescence intensity. After the protection by CNPs, the red fluorescence intensity gradually became stronger and the green fluorescence intensity gradually decreased, and the fluorescence ratio gradually increased with the increase of CNPs concentration. CNPs significantly improved the depolarization of the mitochondrial membrane.

Then the organelle was directly observed with TEM after exposing to oxalate or the CNP intervention, and the results are shown in Fig. 3E. The cells in the normal group were long shuttle-shaped under low magnification, with intact cell membranes and no swelling and collapse of cells; all organelles were visible intact under high magnification, especially the mitochondria without swelling; the inner and outer mitochondrial membranes were intact, and the mitochondrial cristae were clearly visible without collapse; very few autophagosomes were occasionally seen. On the contrary, after oxalate damage, the HK-2 cells were rounded and wrinkled under low magnification, and the surface of the cell membrane was not smooth with a large number of swollen microvilli; under high magnification, the mitochondria were swollen, the mitochondrial bilayer membrane structure was blurred, and the number of mitochondrial cristae was reduced and disordered; at the same time, a large number of autophagosomes were visible, and a large number of swollen microvilli were seen on the surface of the cell membrane. After 24 h of protection by 12.5 µg/mL CNPs, the overall morphology of the cells was close to that of normal cells without wrinkles; under high magnification, there was no swelling of mitochondria, the inner and outer mitochondrial membranes were intact, and the mitochondrial cristae were clear without collapse; autophagosomes were occasionally seen. The results show that CNPs could help protect the mitochondria from oxalate damage.

In order to further investigate whether CNPs could affect the cell cycle of the HK-2 cells or not. The cell cycle progression of damaged HK-2 cells treated with CNPs was detected by flow cytometry (Fig. 3F, G). After treatment of HK-2 cells with 1 mM oxalate, there was an increase in G0–G1 phase cells (percentage increased from 45.08 to 64.14%), a decrease in S phase cells (percentage decreased from 34.45 to 27.26%), and a decrease in G2M phase cells (percentage decreased from 20.47 to 8.6%) compared with the control group. HK-2 cells stagnated in the G0–G1 phase (the percentage decreased from 20.47 to 8.6%), indicating that oxalate injury caused HK-2 cells to stagnate in the G0-G1 phase. After 24 h of protection of damaged HK-2 cells by CNPs, cells blocked in the G0–G1 phase were relieved, with significant remission of cycle block in the 12.5 µg/mL CNPs group, where the percentage was reduced from 64.14 to 52.49% compared with the oxalate damaged group, and the percentage of G/M and S phase cells increased and converged to the normal group. This indicates that CNPs can attenuate the damage caused by oxalate on the DNA of HK-2 cells, thus reducing the blockage of cell cycle progression by oxalate and showing a better protective effect on HK-2 cells.

### Mechanism study of CNPs in suppresses kidney calcium oxalate crystal depositions

**Fig. 4:**
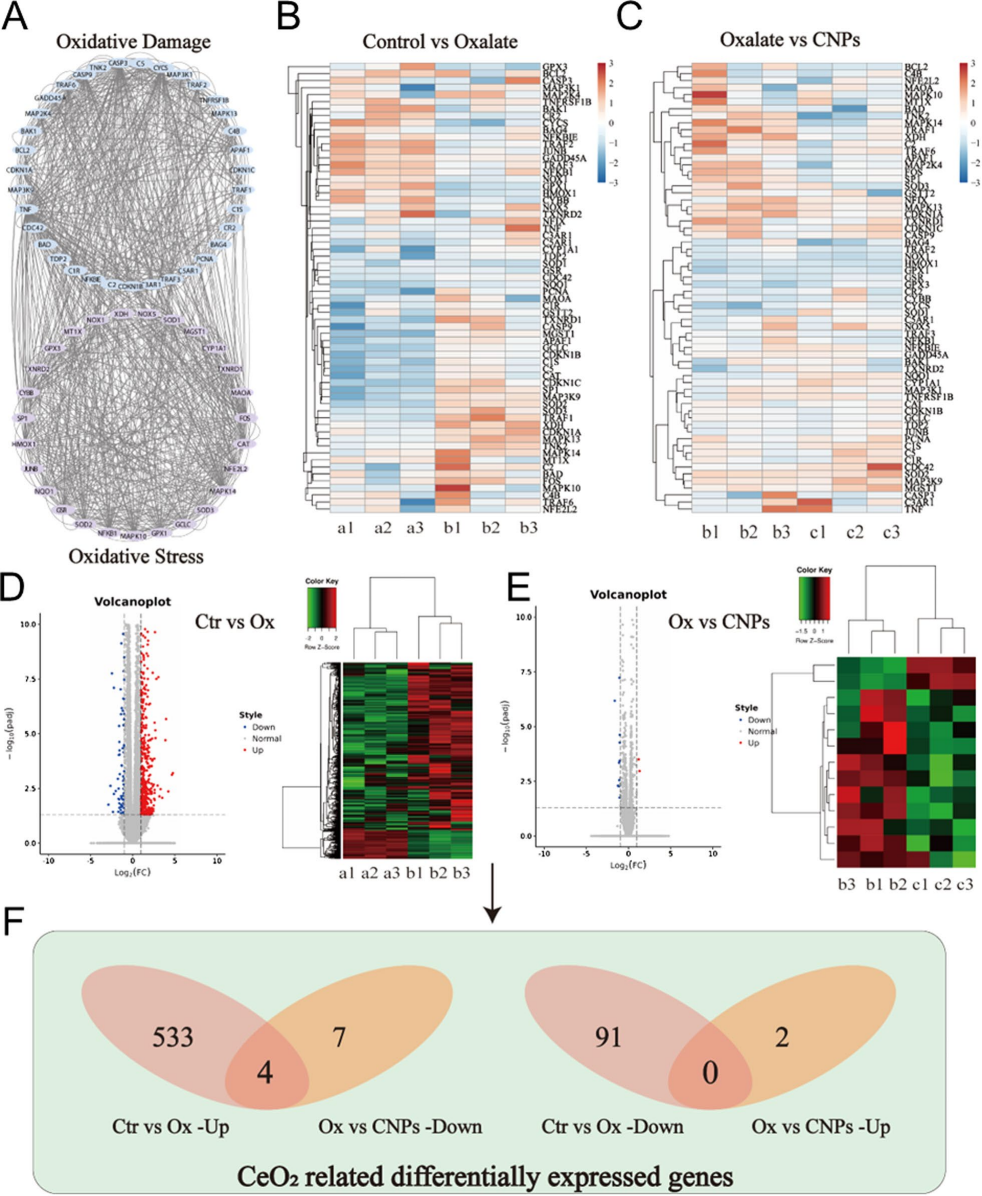
Mechanism study of CNPs in suppresses kidney calcium oxalate crystal depositions.** A** 34 oxidative stress and 39 oxidative damage-related genes from the GSEA database. **B**, **C** The heatmap indicates that the expression of genes related to oxidative stress and oxidative damage; **D**, **E** Volcano plot analysis between different groups. The volcano plot was constructed using the fold change values and P-adjust. Red dots indicate upregulated genes; blue dots indicate downregulated genes. **F** Venn diagram analyzed of the CeO_2_-related differential gene expression between different groups. (Left Venn diagram: the left pale indicated number of up-regulated genes in the oxolate group versus the control group, and the right pale indicated number of down-regulated genes in the CNP intervention group versus the oxolate group; Right Venn diagram: the left pale indicated number of down-regulated genes in the oxolate group versus the control group, and the right pale indicated number of up-regulated genes in the CNP intervention group versus the oxolate group, and overlapped to obtain the intersection.)

In order to further seek the possible mechanism of CNPs on oxidative stress injury of renal epithelial cells induced by high oxalate and suppresses kidney calcium oxalate crystal depositions, cell samples were collected for high-throughput sequencing of mRNA. We first obtained 34 oxidative stress and 39 oxidative damage-related genes from the GSEA database (Fig. 4A). The heatmap revealed that oxidative stress and oxidative damage were significantly differentially expressed between the control and modeling groups and between the modeling and treatment groups (Fig. 4B, C). The upregulated genes in the oxalate group compared with the control group, and the down-regulated genes in the CNP intervention group compared with the oxalate group were shown in Fig. 4D, E. In order to further explore the differentially expressed genes related to the anti-calculus effect of CNPs, we screened differentially expressed genes between up-regulated genes in the oxalate group versus the control group and down-regulated genes in the CNP intervention group versus the oxalate group or opposing situation in Fig. 4F, and obtained four differentially expressed genes related to the anti-calculus effect of CNPs: ENG, MYCBPAP, SPTBN5, ITGA10. It should be noted that ITGA10 is involved in cell adhesion and cell surface-mediated signal transmission. Oxidative stress injury in renal epithelial cells leads to cell membrane rupture and promotes the upregulation of crystal binding molecules, thereby promoting crystal adhesion to cell membranes. Therefore, we speculate that ITGA10 may be a key gene which could express protein involved in the adhesion of renal epithelial cells through cell surface-mediated signal transmission, promote the adhesion of crystals, and ultimately lead to the formation of stones, which needs to be further verified by our follow-up experiments. The relationship between ENG, MYCBPAP, SPTBN5 and anti-calculus has not yet been reported and needs to be further explored.

### Effects of CNPs in EG-induced rat’s kidney pathological alterations

The in vitro experimental results showed that CNPs could significantly inhibit the oxidative stress damage induced by high oxalate in renal epithelial cells. In order to further explore the antioxidant and anti-stone abilities of CNPs in vivo, an ethylene glycol-induced rat kidney calcium oxalate crystal deposition model was constructed.

To further verify the distribution of cerium oxide in mice, we first detected the distribution and metabolism of the four CNPs in nude mice at 0, 1, 2, 4, 8, 12 h using a living animal imager. The results in Additional file 1: Fig. S4 showed that after gavage, CeO_2_ with different shapes was first accumulated in the stomach, metabolized to the kidney at 2 h, and metabolized to the bladder at 8 h, after 12 h, it was almost metabolized out of the body, indicating that CeO_2_ can effectively prevent the formation of kidney stones. In addition, this result also indicates that CeO_2_ can effectively eliminate the body in a short period of time, without causing cumulative toxicity. According to previous study [42, 43], the bioavailability of CNP may be higher than 30% at 14 days.

**Fig. 5:**
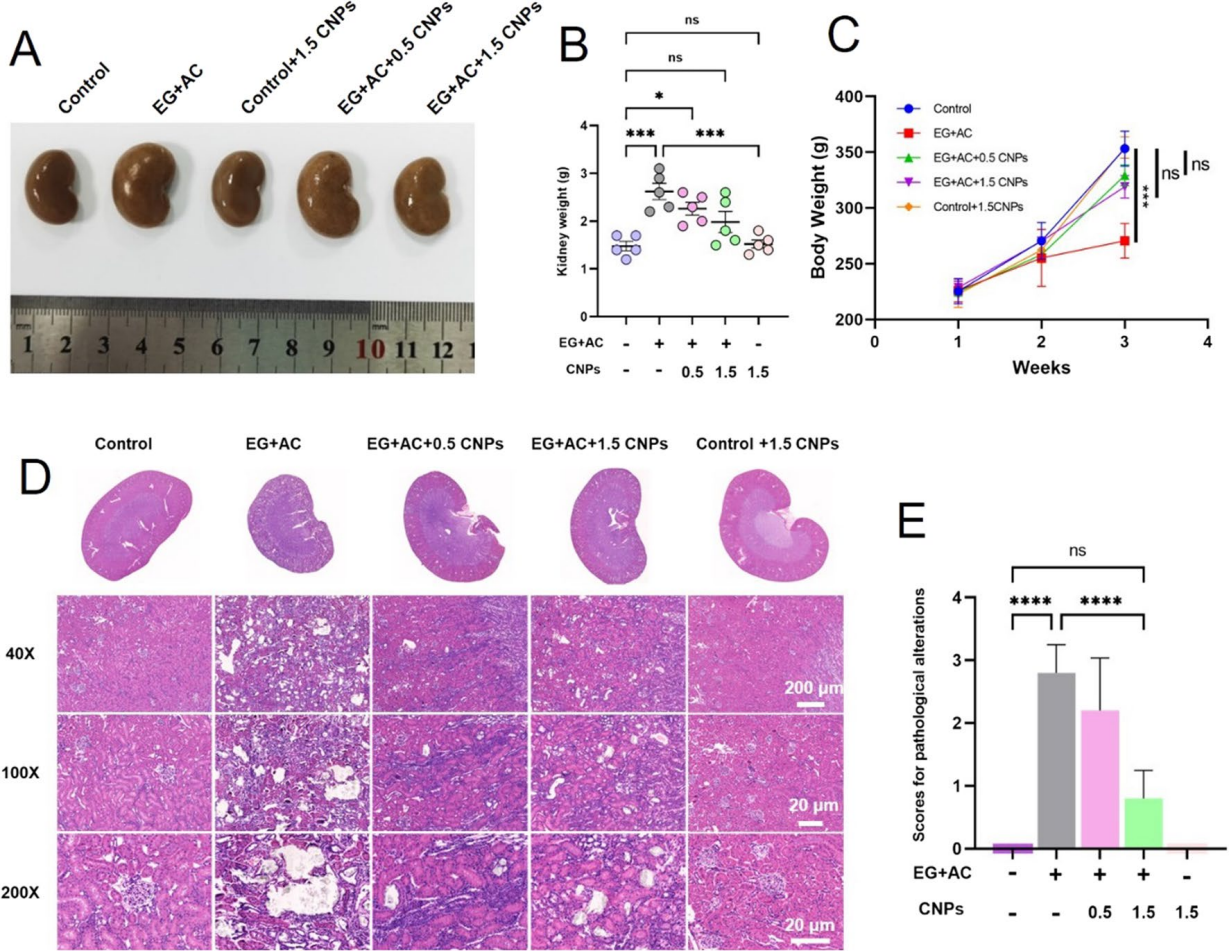
Effects of CNPs in EG-induced rat’s kidney pathological alterations.** A**, **B** The gross anatomy of rat’s kidney and the kidney weight of each group of rats; **C** shows the weight change of each group of rats during the experiment; **D**, **E** H&E staining was used to assess renal pathological alterations and the degree of renal injury in each group of rats. Magnification is ×40, Scale bar = 200 μm; ×100, Scale bar = 20 μm; ×400, Scale bar = 20 μm; * indicates P < 0.05, *** indicates P < 0.001, **** indicates P < 0.0001, ns indicates P > 0.05, no significant difference

The results in Fig. 5A revealed that the gross anatomy of the rat kidney, the rats in the control group conformed to the size of the kidney at that week of age, with good morphology, and the kidney size in the control + 1.5 CNPs group and EG + AC + 1.5 CNPs group was close to the normal group, with no significant difference while the tissue volume of the kidney of the rats in the EG + AC group and EG + AC + 0.5 CNPs group was significantly increased, which might be caused by the damaged kidneys and reduced renal function in rats after modeling, resulting in edema of kidney tissues and deposition of CaOx crystals, which eventually led to larger kidney size. The trend of the kidney weight is consistent with the kidney size, and the results are shown in Fig. 5B. What’s more, the result in Additional file 1: Fig. S5 showed that CNPs have excellent biosafety in vivo since rat heart, liver, spleen, and lung tissues are not innocuous to them.

Figure 5C shows the changes of the body weight of rats in each group during the experiment, the body weight of rats in each group increased with the increase of the rat’s week age, among which the body weight of control group, control + 1.5 CNPs group, EG + AC + 0.5 CNPs group and EG + AC + 1.5 CNPs group was significantly higher than that of EG + AC group. The results showed that there was no significant difference between the EG + AG + 1.5 CNPs group and the EG + AG + 0.5 CNPs group, but there was a significant difference between the model group and the control group, indicating that the body weight of rats was significantly reduced after modeling, while CNPs intervening had no effect on the body weight of the rats. This result indicates that CNPs have no significant toxicity to mice.

H&E staining of the kidney tissues of rats in each group was shown in Fig. 5D. After managing with EG + AC, a large number of calcium oxalate crystals were deposited in the paraglomerular, intertubular and tubular lumen of rats, and the distribution was found in the renal cortex and renal medulla, and structural damage was seen in the kidneys of rats, such as dilatation, destruction, discontinuity and even fracture of the tubules and detachment of epithelial cells. The amount of calcium oxalate crystals deposited in the kidney and the destruction of renal tubules were significantly reduced with the increase of CNPs concentration, among which the EG + AC + 1.5 CNPs group had the best effect. We also compared the scavenging capacity and protective effect of four kinds of CNPs on ROS in rats. Through H&E staining and polarizing microscope observation of the kidney, the results in Additional file 1: Fig. S6 showed that the crystal of the porous nanorods CeO_2_ group was significantly lower than that of the control group compared with other groups. This result showed that the porous nanorods CeO_2_ presents the best antioxidant and anti-calcium oxalate crystal deposition ability in vivo.

The pathological scores of kidney injury in each group of rats, tubular dilatation and inflammatory cell infiltration were the main scoring references, as shown in the Fig. 5E, EG-induced modeling group damage scores were significantly higher than the control group; after 0.5 and 1.5 mg/kg.wt CNPs protected the kidney, the damage scores were decreased. Among them, the EG + AC + 1.5 CNPs group was significantly different from the EG-modeling group. CNPs significantly inhibited EG-induced renal pathological alterations in rats, which may be related to their strong antioxidant stress damage ability.

### Effects of CNPs in EG-induced CaOx crystals depositions in rat kidneys

**Fig. 6:**
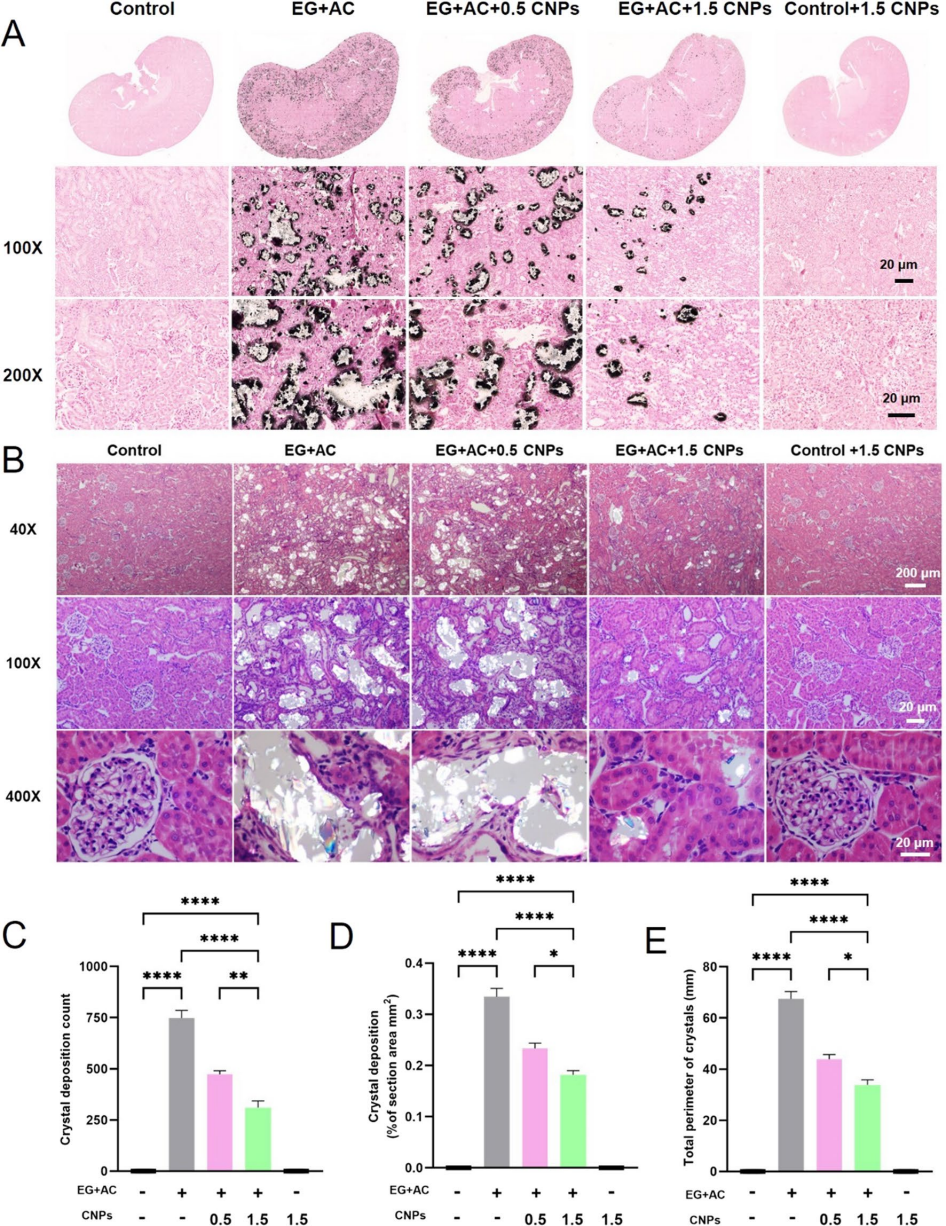
Effects of CNPs in EG-induced CaOx crystals depositions in rat kidneys.** A** Pizzolato staining of the calcium oxalate crystal deposition in the kidney of rats. **B–E** The distribution of crystals and crystal deposition statistics of each group of rat kidney sections observed under polarized light microscope (Magnification is ×40, Scale bar = 200 μm, ×100, Scale bar = 20 μm, ×400, Scale bar = 20 μm; * indicates P < 0.05, ** indicates P < 0.01, and **** indicates P < 0.0001)

To verify the inhibitory ability of CNPs on EG-induced intrarenal calcium oxalate crystal deposition in rats, we subjected rat kidneys to further assays. The results showed that the observation of calcium oxalate crystal deposition in the kidney of rats under Pizzolato staining was shown in Fig. 6A, the areas with calcium oxalate crystals were stained black or brown-black, no crystal deposition was seen in the kidney of rats in the control group and control + 1.5 CNPs group, and a large number of black or brown-black crystals were deposited in the cortex and medulla of rats in the EG + AC group. The crystal deposition in the EG + AC + 0.5 CNPs group was similar to that in the modeling group, while in the EG + AC + 1.5 CNPs group, the crystal deposition in the kidney was significantly reduced compared with that in the modeling group.

Figure 6B, C shows the distribution of crystals and crystal deposition statistics of each group under a polarized light microscope. The kidneys of the control group and control + 1.5 CNPs group conformed to the size and morphology of the growth period, and the structures of tubules and glomeruli were normal. A large number of strongly refractive calcium oxalate crystals could be seen in the EG + AC group under low magnification in the renal cortex and renal medulla. In the EG + AC + 0.5 CNPs group, crystal deposition and tubular destruction were similar to those in the EG + AC group, while in the EG + AC + 1.5 CNPs group, crystal deposition and tubular destruction were significantly reduced compared with those in the modeling group.

The number of crystals, the total area and circumference of crystals under the field of view were calculated by randomly selecting three fields of view in each group of kidney sections and shown in Fig. 6C–E. Compared with the kidney crystal situation in the control group, the kidney calcium oxalate crystal deposition(Fig. 6C), the total the total area (Fig. 6D) and the circumference of crystals (Fig. 6E) in the EG + AC group was significantly increased in both the number of crystals. In contrast, after 0.5 and 1.5 mg/kg.wt CNPs intervention in the modeling process, the amount of calcium oxalate crystals deposited in the kidney and the destruction of renal tubules were significantly reduced with the increase of CNPs concentration. CNPs significantly inhibit hyperoxalate-induced calcium oxalate crystal deposition in rat kidney, demonstrating its strong anti-stone ability.

### Effects of CNPs on EG-induced renal oxidative injury in rat kidneys

Previous studies have shown that CNPs have a significant inhibitory effect on pathological damage in rat kidney. We hypothesized that CNPs might inhibit oxidative stress injury induced by hyperoxalate, and to further verify our speculation, we conducted further studies.

**Fig. 7:**
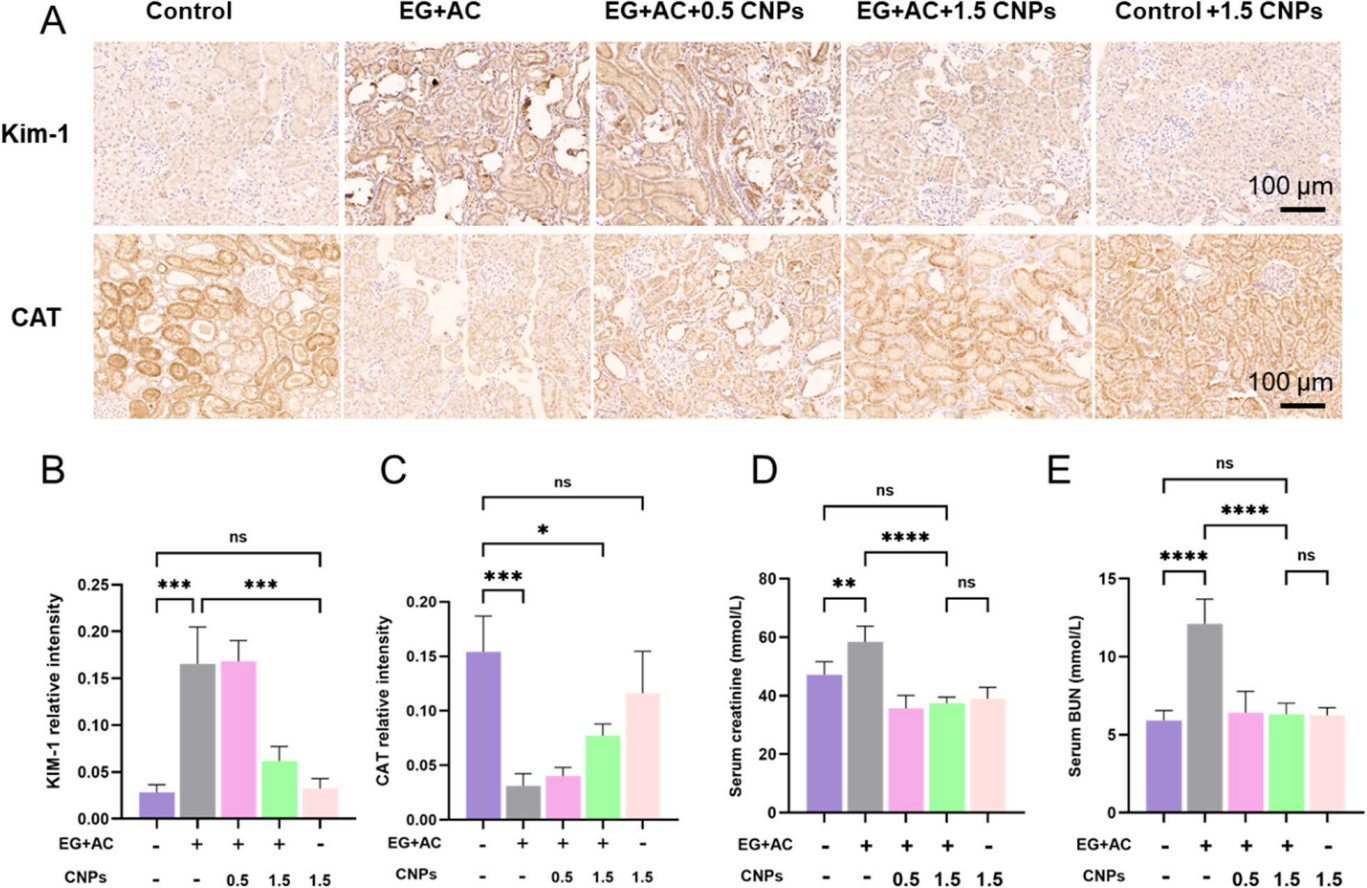
Analysis of tissue and urine indicators of different groups.** A** Protein expression of KIM-1, CAT by IHC assay in different treatment groups. **B**, **C** are semi-quantitative statistical plots of the expression of KIM-1 and CAT in the kidney of rats and their mean optical density detected by immunohistochemistry. **D–F**) Indicates the levels of creatinine and BUN in the serum of each group of rats; Magnification is ×200, Scale bar = 100 μm;** indicates P < 0.01, *** indicates P < 0.001, and **** indicates P < 0.0001, ns indicates P > 0.05, no significant difference

Kidney injury molecule (KIM-1) is a type I transmembrane protein expressed in damaged proximal tubular epithelial cells. KIM-1 can be used as a highly sensitive and specific biomarker for the diagnosis of early kidney injury. In the present study, the expression level of KIM-1 in the modeling group induced by ethylene glycol was significantly up-regulated compared with the control group. After the intervention of the CNPs group in the modeling process, the expression level of KIM-1 in the kidney gradually decreased, in which the expression level of KIM-1 in the EG + AC + 1.5 CNPs group was significantly down-regulated compared with the EG + AC group (P < 0.0001). Ethylene glycol can cause renal injury and significantly upregulate the expression level of KIM-1 in rat kidney, while CNPs intervention in this modeling process can significantly reduce the damaging effect of modeling on rat kidney, which has a significant protective effect on kidney (Fig. 7A, B).

Catalase (CAT) is one of the key antioxidant enzymes in biodefense system. Compared with the control group, the expression of CAT in rat kidney tissues was significantly down-regulated in the ethylene glycol-induced modeling group, while the expression level of CAT in the kidney was gradually restored to normal after CNPs intervention, in which the expression level of CAT in the EG + AC + 1.5 CNPs group was restored more significantly (p < 0.0001). Therefore, CNPs could inhibit ethylene glycol-induced oxidative stress damage in rat kidney and maintain the homeostasis of intracellular antioxidant levels in the rat kidney (Fig. 7A, C).

Serum CRE and BUN are important indicators to assess the damage to the kidney and renal tubules. Due to the presence of many crystal deposits in the kidneys of rats modeled with EG + AC, which impeded the excretion of urine and metabolic wastes, especially nitrogenous substances, the renal function of the rats was affected, resulting in a significant decrease in impaired glomerular filtration rate (GFR). Compared with the control group, the serum CRE and BUN levels in the modeled rats were significantly increased (Fig. 7D, E). After the intervention of CNPs, the serum CRE and BUN levels in the rats showed a decreasing trend, indicating that CNPs could significantly reduce the EG-induced renal injury and had a protective effect on renal function. The 24 h urine and serum ion biochemical indexes of rats in Additional file 1: Fig. S7 showed no significant difference in each group.

## Conclusion

In summary, we used porous nanorods CeO_2_ nanozyme to catalyze the decomposition of excess free radicals and acts as an antioxidant reagent to suppress kidney calcium oxalate crystal depositions due to the reversible transformation from Ce^3+^ to Ce^4+^. It was found that due to their large specific surface area and strong ability to scavenge free radicals, CNPs can effectively reduce the production of reactive oxygen species, restore the polarity of mitochondrial membrane potential, restore cell cycle progression, reduce cell death, and inhibit the formation of calcium oxalate crystals on the cell surface. The results of high-throughput sequencing of mRNA showed that CNPs could protect renal epithelial cells from oxidative stress damage caused by high oxalate through suppressing the expression gene of cell surface adhesion proteins. In addition, CNPS significantly reduced pathological tubular injury and inhibited calcium oxalate crystal deposition in rats without significant side effects on other organs and physiological indicators in vivo. Our results provide a new strategy for CNP_S_ as a potential for clinical prevention of crystalline kidney injury and crystal deposition.

## Supplementary Information

**Additional file 1: Fig. S1. **A) XRD of porous nanorods, nanorods, nanopolyhedra andnanocubes CeO_2_; B) FTIR of porous nanorods, nanorods,nanopolyhedra and nanocubes CeO_2_; C, D) XPS and XPS spectra of Ce3d_5/2_and Ce3d_3/2_ of porous nanorods,nanorods, nanopolyhedra and nanocubes CeO_2_.** Fig. S2.** A) BET analysis of different morphologies of CeO_2_, inset is the TEM of the as-prepared CeO_2_; B) ROS levels of HK-2 cells after treated by different morphologiesof CeO_2_. **Fig. S3.** A–D)Effect of different concentrations of CNPs on the viability of HK-2 cells, NRK-49F, NRK-52E, and MDCK cells. ns indicates nostatistical difference compared with the control group, P > 0.05. **Fig. S4.** In vivo biodistributionstudies of four different types of ICG labelednano ceria (200 μL 1.5 μg/mL/kg.wt CNPs solution) in nude mice at 0, 1, 2, 4, 8, 12 hours using a living animal imager. **Fig. S5.** A, B) H&E staining of rat heart, liver, spleen, and lung tissues, A: magnification ×10; B: magnification×100, Scale bars = 200 μm. **Fig. S6.** H&E staining (A) and polarizing microscope observation (B) of the kidney before and after four kindsof CNPs treatment. **Fig. S7.** The 24 h urine andserum ion biochemical indexes of rats in each group.
