# Supplementary material for: Cerium oxide-based nanozyme suppresses kidney calcium oxalate crystal depositions via reversing hyperoxaluria-induced oxidative stress damage
Source: J Nanobiotechnology. 2022 Dec 8;20:516. doi: 10.1186/s12951-022-01726-w (PMC9733203; doi:10.1186/s12951-022-01726-w)
Supplement: Supplementary file 1 — Additional file 1: Fig. S1. A) XRD of porous nanorods, nanorods, nanopolyhedra andnanocubes CeO2; B) FTIR of porous nanorods, nanorods,nanopolyhedra and nanocubes CeO2; C, D) XPS and XPS spectra of Ce3d5/2and Ce3d3/2 of porous nanorods,nanorods, nanopolyhedra and nanocubes CeO2. Fig. S2. A) BET analysis of different morphologies of CeO2, inset is the TEM of the as-prepared CeO2; B) ROS levels of HK-2 cells after treated by different morphologiesof CeO2. Fig. S3. A–D)Effect of different concentrations of CNPs on the viability of HK-2 cells, NRK-49F, NRK-52E, and MDCK cells. ns indicates nostatistical difference compared with the control group, P > 0.05. Fig. S4. In vivo biodistributionstudies of four different types of ICG labelednano ceria (200 μL 1.5 μg/mL/kg.wt CNPs solution) in nude mice at 0, 1, 2, 4, 8, 12 hours using a living animal imager. Fig. S5. A, B) H&E staining of rat heart, liver, spleen, and lung tissues, A: magnification ×10; B: magnification×100, Scale bars = 200 μm. Fig. S6. H&E staining (A) and polarizing microscope observation (B) of the kidney before and after four kindsof CNPs treatment. Fig. S7. The 24 h urine andserum ion biochemical indexes of rats in each group. [file 12951_2022_1726_MOESM1_ESM.docx]

**Supplementary Material**

**Cerium oxide-based** **nanozyme suppresses kidney calcium oxalate crystal depositions *via* reversing hyperoxaluria-induced oxidative stress damage**

Jiwang Deng^1#^, Bangxian Yu^1#^, Zhenglin Chang^1^, Sicheng Wu^1^, Guanlin Li^1^, Wenzhe Chen^1^, Shujue Li^1^, Xiaolu Duan^1^, Wenqi Wu^1,2^, Xinyuan Sun^1*^, Guohua Zeng^1*^, Hongxing Liu^1*^

1. Department of Urology, Guangzhou Institute of Urology, Guangdong Key Laboratory of Urology, the First Affiliated Hospital of Guangzhou Medical University, Guangzhou Medical University, Guangzhou, China;

2. Department of Urology, the Second Affiliated Hospital of Guangzhou Medical University, Guangzhou Medical University, Guangzhou, China

*Correspondence:

Xinyuan Sun: [sunxinyuan1985@163.com](mailto:sunxinyuan1985@163.com)

Guohua Zeng: [2008690094@gzhmu.edu.cn](mailto:2008690094@gzhmu.edu.cn)

Hongxing Liu: [liuhongxing@gzhmu.edu.cn](mailto:liuhongxing@gzhmu.edu.cn)

^#^These authors have contributed equally to this work

**
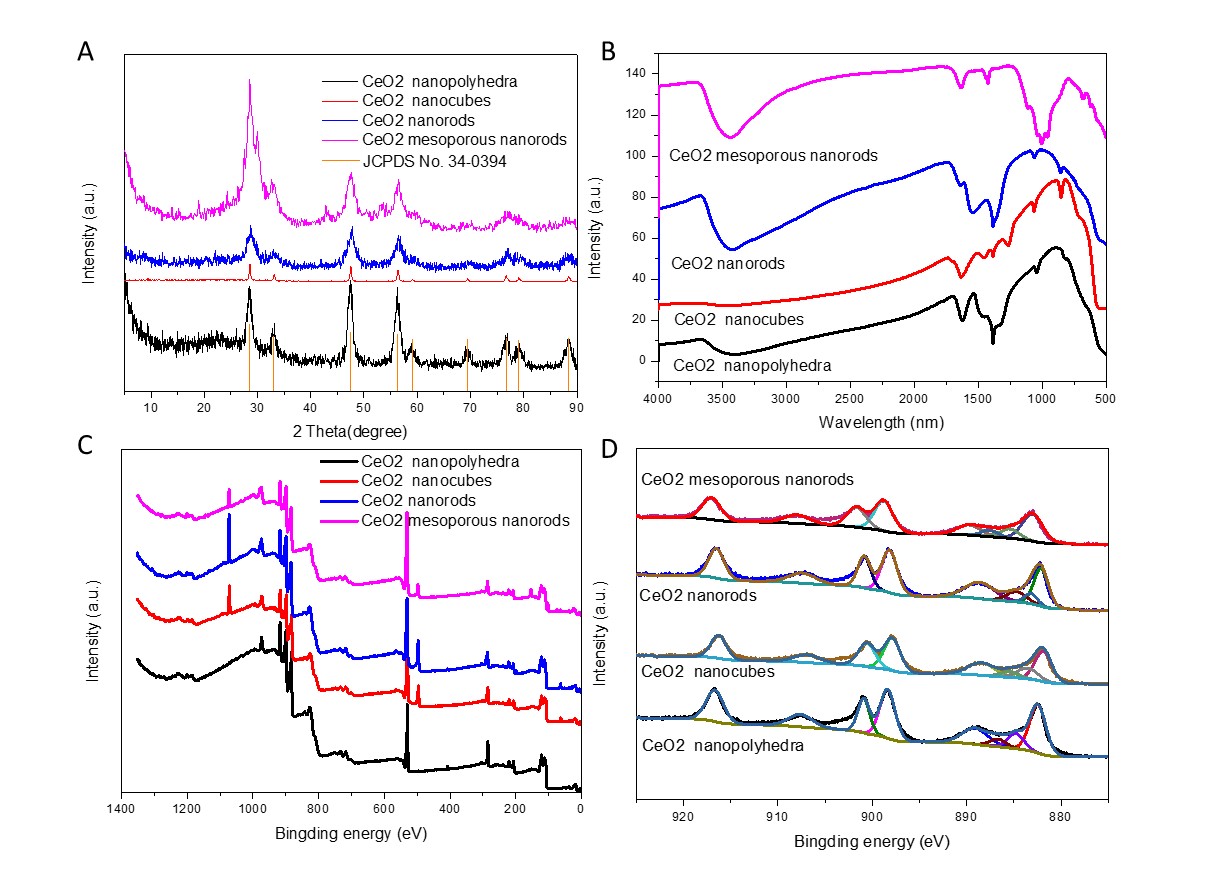
 Fig.S1 A)** XRD of porous nanorods, nanorods, nanopolyhedra and nanocubes CeO_2_; **B)** FTIR of porous nanorods, nanorods, nanopolyhedra and nanocubes CeO_2_; **C-D)**XPS and XPS spectra of Ce3d_5/2_ and Ce3d_3/2_ of porous nanorods, nanorods, nanopolyhedra and nanocubes CeO_2._


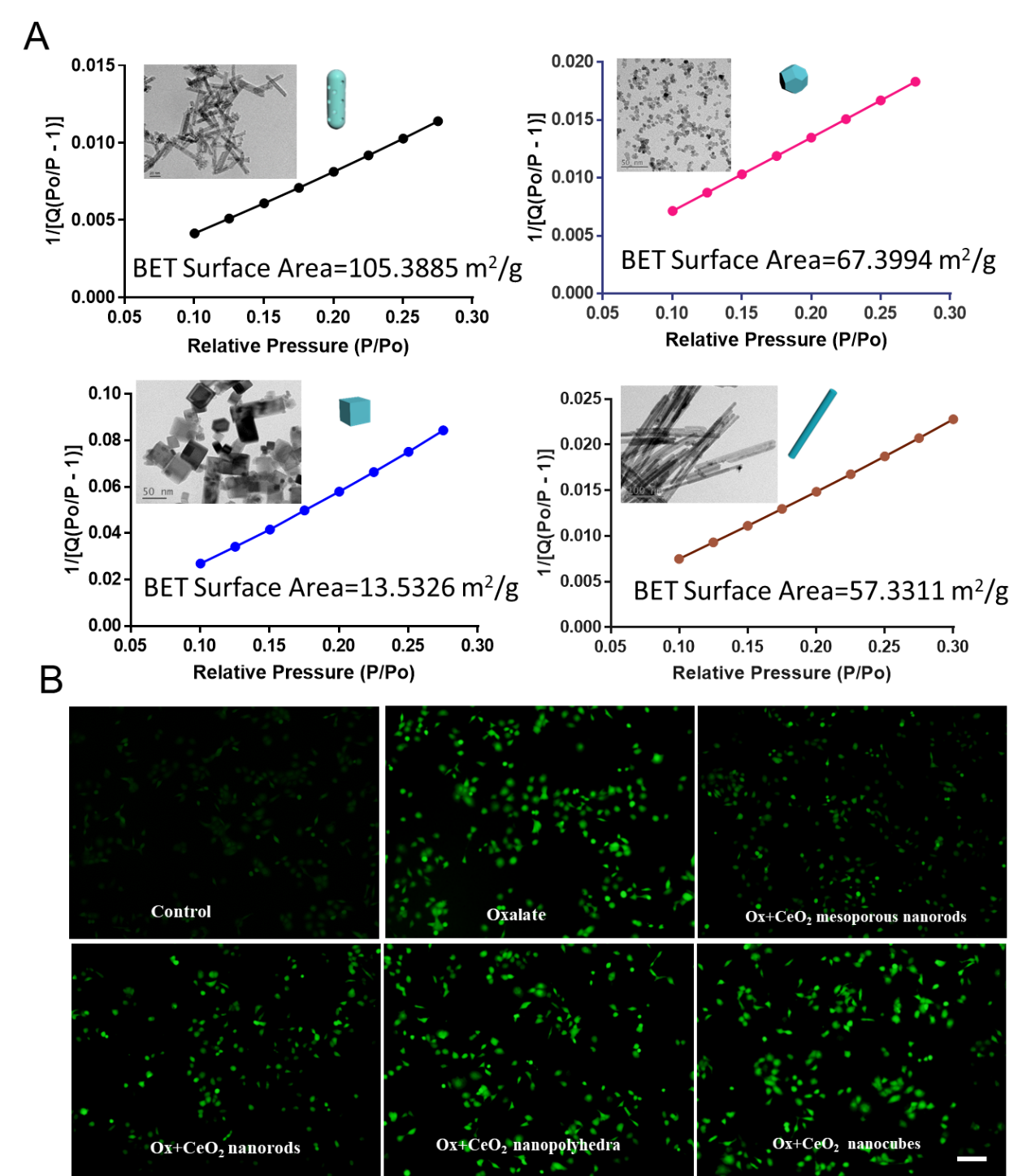


**Fig.S2** **A)** BET analysis of different morphologies of CeO_2_, inset is the TEM of the as-prepared CeO_2_; **B)** ROS levels of HK-2 cells after treated by different morphologies of CeO_2._


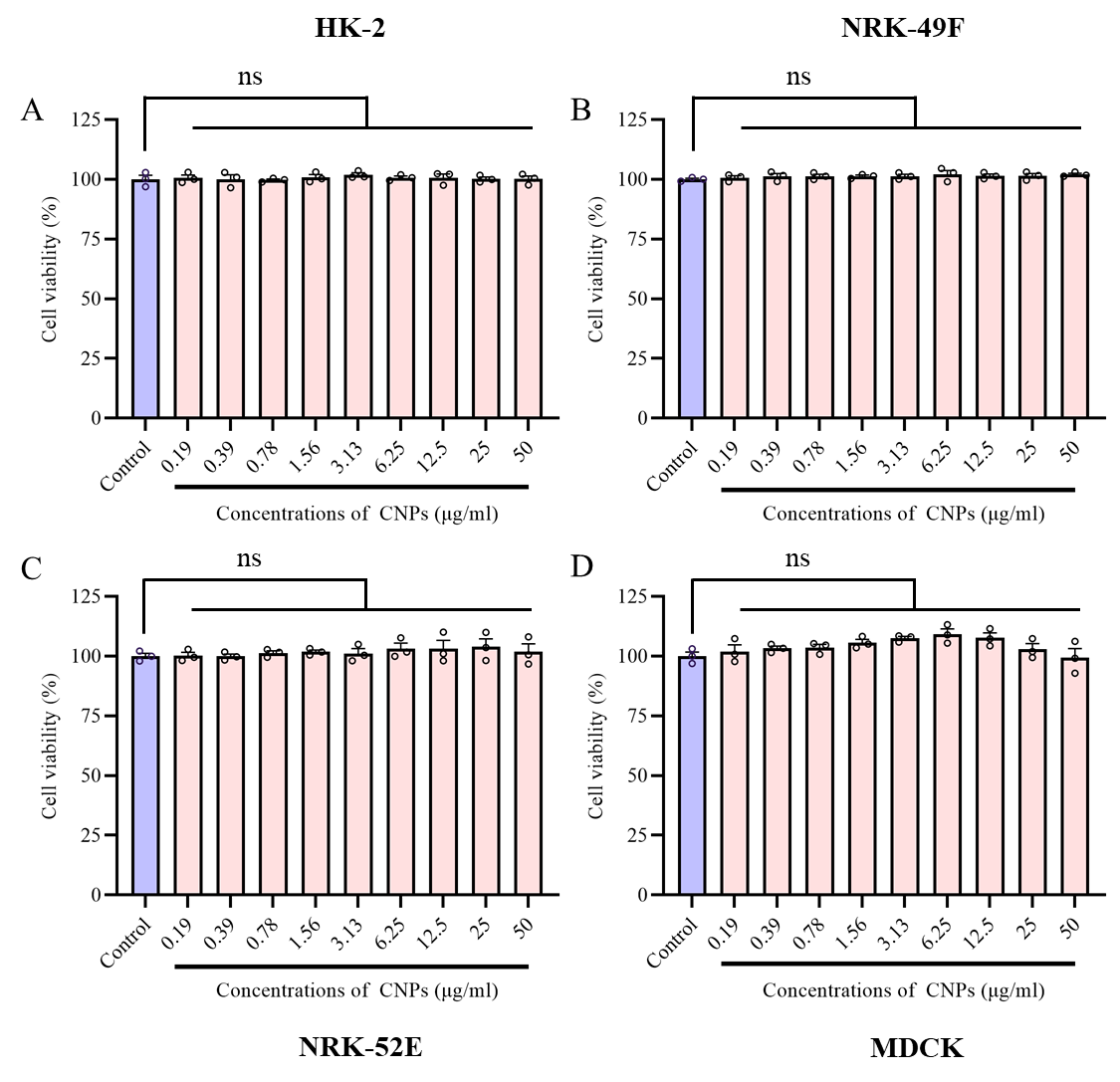


**Fig.S3 A-D)** Effect of different concentrations of CNPs on the viability of HK-2 cells, NRK-49F, NRK-52E, and MDCK cells. ns indicates no statistical difference compared with the control group, P > 0.05. *
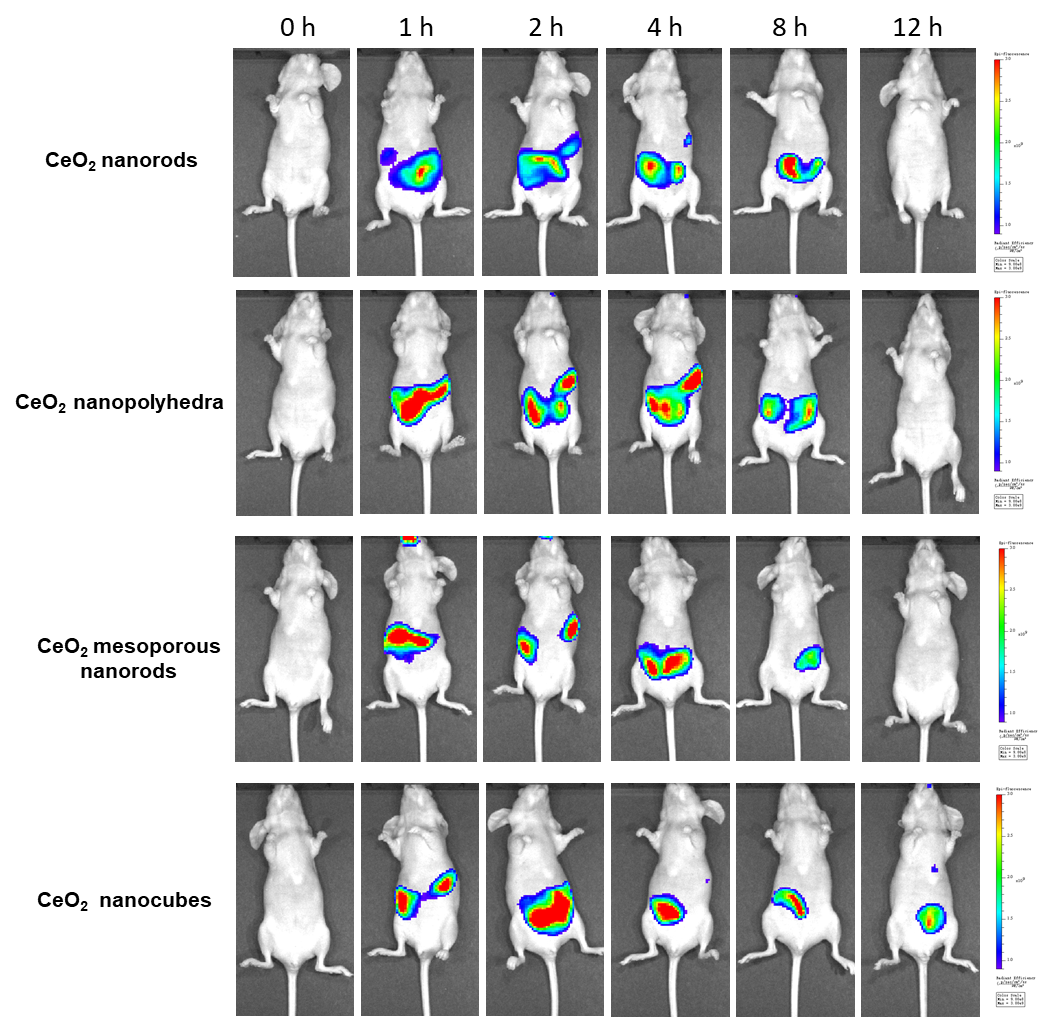
*

**Figure S4** In vivo biodistribution studies of four different types of ICG labeled nano ceria (200 μL 1.5 μg/mL/kg.wt CNPs solution) in nude mice at 0, 1, 2, 4, 8, 12 hours using a living animal imager.


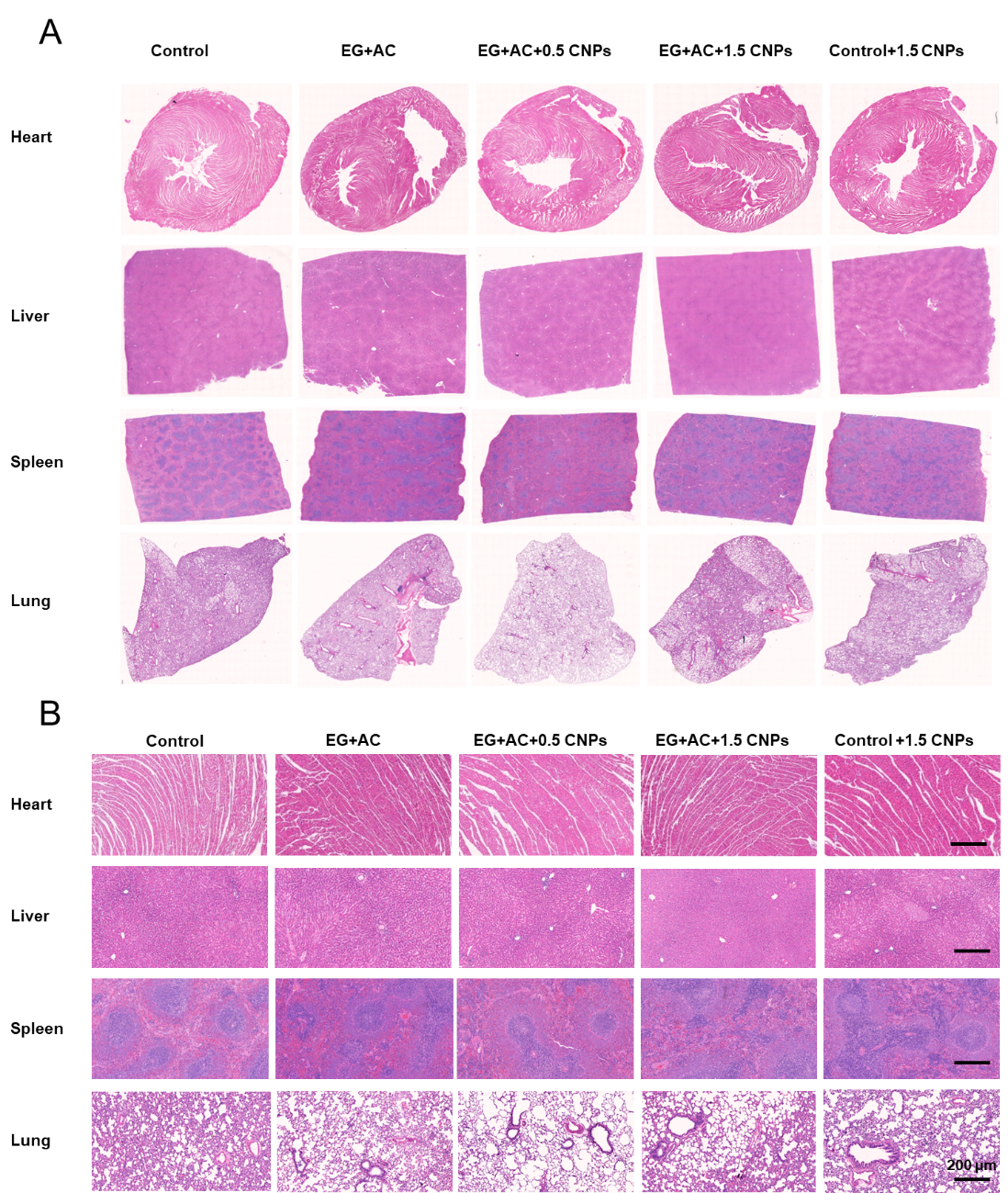


**Fig.S5 A-B)** H&E staining of rat heart, liver, spleen, and lung tissues, A: magnification 10x; B: magnification 100x, Scale bars=200 μm.

**
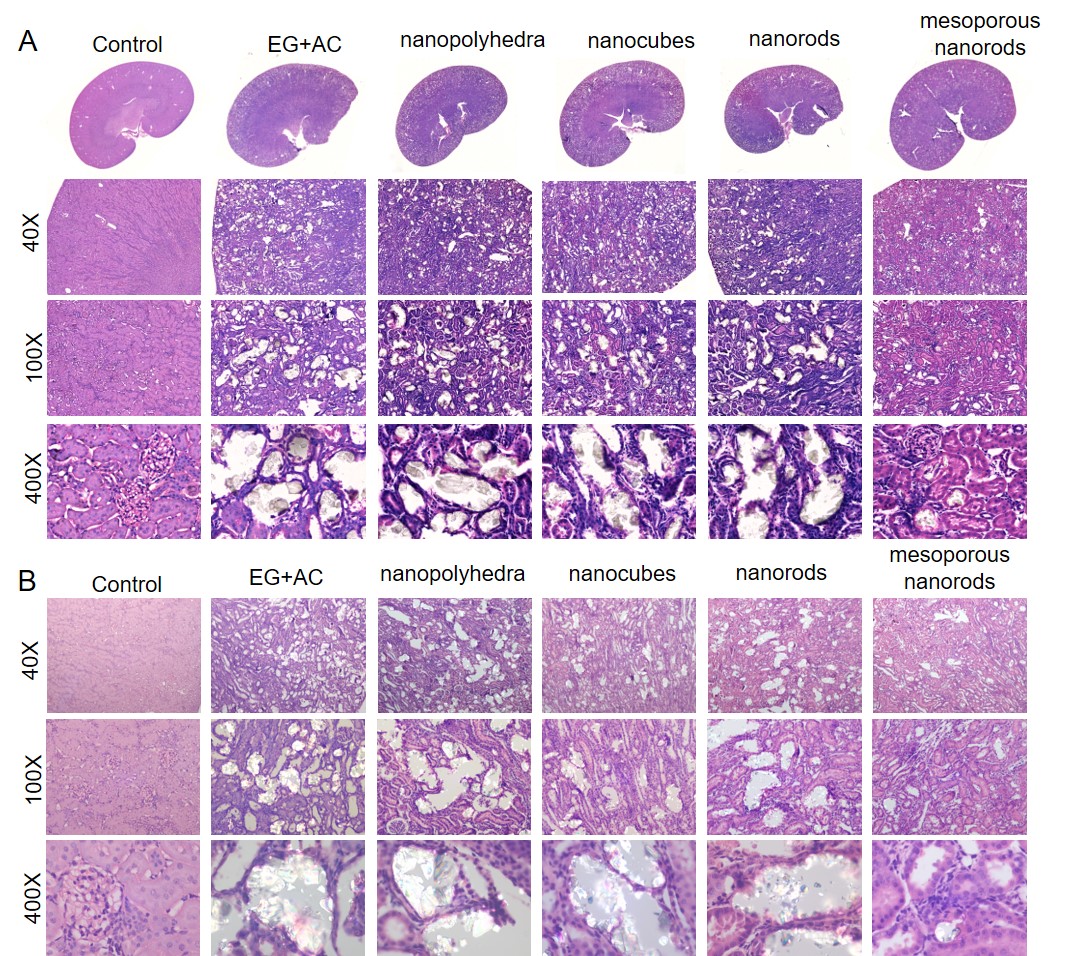
**

**Fig.S6** H&E staining(A) and polarizing microscope observation(B) of the kidney before and after four kinds of CNPs treatment.

**
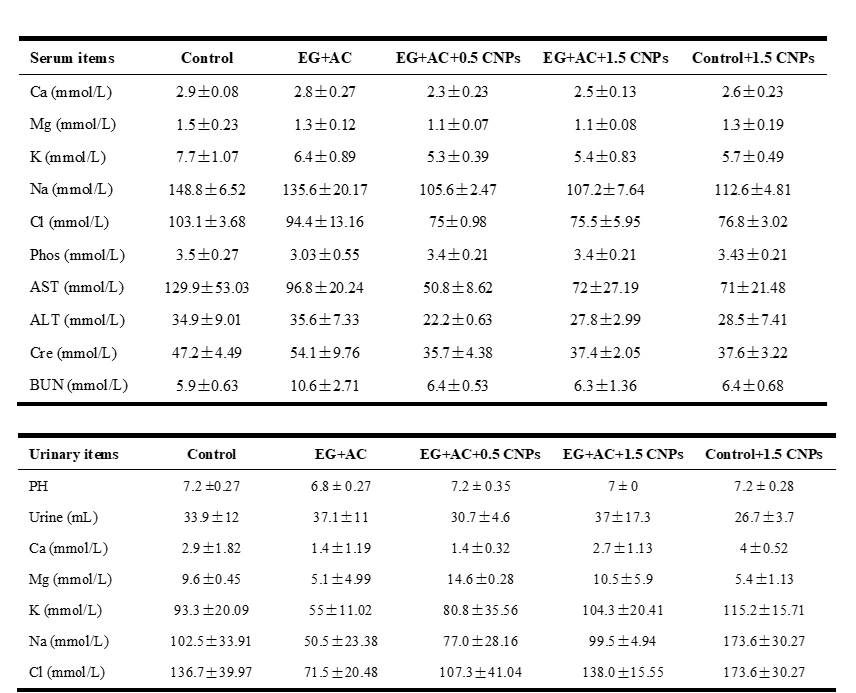
**

**Fig.S7** The 24 h urine and serum ion biochemical indexes of rats in each group
